# Home-based sleep monitoring using a novel non-contact, radar-based biomotion sensor in adults with cystic fibrosis treated with elexacaftor/tezacaftor/ivacaftor

**DOI:** 10.1007/s11325-025-03451-8

**Published:** 2025-09-23

**Authors:** Sarah Dietz-Terjung, Svenja Straßburg, Christoph Schöbel, Tim Schulte, Paul Dietz, Fatma Ezzahra Gahbiche, Jose Ortiz, Christian Taube, Gerhard Weinreich, Sivagurunathan Sutharsan, Matthias Welsner

**Affiliations:** 1 Department of Pulmonary Medicine, University Hospital Essen-Ruhrlandklinik, University of Duisburg-Essen, Essen, Germany; 2 Department of Sleep and Telemedicine, University Hospital Essen - Ruhrlandklinik, University of Duisburg-Essen, Essen, Germany; 3 Center of interdisciplinary Telemedicine and TeleCare, University Medicine Essen-Ruhrlandklinik, University of Duisburg-Essen, Essen, Germany

**Keywords:** Cystic fibrosis, Sleepiz one++, ETI therapy, Respiratory rate, Sleep monitoring, Infection detection

## Abstract

**Purpose:**

Cystic fibrosis (CF) is a life-limiting autosomal recessive disease often treated with triple CFTR modulator therapy (elexacaftor/tezacaftor/ivacaftor, ETI). Sleep disturbances are common in people with CF (pwCF). This study evaluated the Sleepiz One+, a non-contact radar-based biomotion sensor, for monitoring respiratory rate, heart rate, apnea-hypopnea index (AHI), and sleep quality in pwCF with ≥ 1 F508del allele during ETI therapy. The device’s suitability for home monitoring of therapy success was also assessed.

**Methods:**

In a six-month observational study, 58 adult pwCF (2908 recordings) used the Sleepiz One + at home to collect longitudinal data on respiratory and sleep parameters during ETI therapy.

**Results:**

Significant reductions in respiratory rate and time in bed were observed after starting ETI, with respiratory rate decreases detectable within 1–5 days. Short-term respiratory rate increases corresponded with infections verified by medical records. Heart rate decreased non-significantly, and sleep efficiency improved modestly. AHI slightly decreased overall but increased in some patients, possibly linked to ETI-related weight gain.

**Conclusion:**

Sleepiz One + reliably tracked respiratory and sleep changes during ETI therapy in adult pwCF. It shows promise for early infection detection via respiratory rate monitoring, warranting further investigation.

## Introduction

Cystic fibrosis (CF) is a life-limiting, autosomal recessive disorder caused by mutations in the CFTR gene, leading to multisystem disease, primarily affecting the lungs [1–4].Globally, about 100,000 individuals are affected, including over 7,000 in Germany. Advances in care have increased median survival to 54 years, with more adults than children living with CF since 2008 [5]. CFTR modulators, particularly the triple combination elexacaftor/tezacaftor/ivacaftor (ETI), significantly improve lung function, nutritional status, and quality of life, and reduce pulmonary exacerbations in people with at least one F508del mutation [6–13]. ETI was approved in Europe in 2020 for people aged ≥ 6 years and has transformed CF management [10–13]. Despite these advances, sleep disturbances—including obstructive sleep apnea (OSA), poor sleep quality, and sleep-related breathing disorders—remain prevalent in people with CF (pwCF), negatively affecting health and quality of life [14–22]. However, there is a lack of validated tools for at-home monitoring of sleep and respiratory changes under CFTR modulator therapy [14–21, 23–25]. The Sleepiz One + is a non-contact radar-based biomotion sensor (Class IIa device) that enables contactless measurement of respiratory rate, heart rate, breathing patterns, AHI, sleep duration, and sleep efficiency [24, 26]. It operates at 24 GHz from approximately 50 cm, detecting thoracic motion without being affected by clothing or blankets. Its algorithms use time-series analysis to calculate parameters validated against cardiorespiratory polysomnography [26]. A more detailed description is available in prior validation reports [24, 26]. This prospective, single-center observational study evaluated the utility of Sleepiz One + over six months in 58 adult pwCF with at least one F508del mutation following ETI initiation. The goal was to assess its suitability for home-based monitoring and its ability to detect therapy-related changes in respiratory and sleep parameters. Results indicated early and sustained reductions in respiratory rate and time in bed, with transient increases in respiratory rate often associated with infections. Sleep efficiency showed a slight, non-significant increase, while AHI changes were linked to BMI gain, highlighting the need for individualized follow-up. These findings support the potential of contactless home-based monitoring for assessing treatment effects and sleep health in pwCF.

## Methods

### Study design and population

This prospective, single-center proof-of-concept study enrolled adult pwCF between September 2020 and July 2021.Inclusion criteria were pwCF aged ≥ 18 years who had at least one F508del CFTR gene variant and were therefore eligible for ETI therapy. Participants were required to be in a clinically stable condition and demonstrate willingness and ability to comply with the study protocol. Exclusion criteria included pregnancy, breastfeeding, and/or presence of a pacemaker. The study protocol was approved by the ethics committee of the University Duisburg-Essen (19-8961-BO) and all procedures were performed in accordance with the Declaration of Helsinki.

### Study design and experimental procedures

The Sleepiz One + device was used to collect data on nocturnal home heart rate, respiratory rate, sleep metrics and the AHI for at least six months after the initiation of ETI therapy.

At the beginning of the study (T0), a biocalibration was carried out for each patient before ETI was administered, i.e. the Sleepiz One + was compared with in-house PSG. The patients then received ETI therapy for the first time, were monitored as inpatients for three days and then discharged to their home environment. After six months (T1), the patients returned the device as part of their routine visit to our university outpatient clinic.

The pwCF were asked to switch on the Sleepiz One + device before bedtime and to switch it off in the morning.

### Data analyses

To be considered for analysis, recordings had to be of minimum signal quality for at least 120 min per night. Respiration rate and heart rate values, as well as the estimations of the AHI, total sleep time (TST) and sleep efficiency (SE) are reported as median values with the 5th and 95th percentile. Other parameters are reported using descriptive statistics (frequency or mean ± standard deviation). The Kolmogorov-Smirnov test was used to test for normal distribution. Sleep-related parameters were assessed using the American Academy of Sleep Medicine guidelines.

Box plots, Sankey plots and Scatter plots were used to visually represent the study data. Boxplots were used to show data at baseline (T0; start of ETI therapy) and after six months of ETI therapy (T1). Values at baseline and 6 months were compared using the paired t-test or Pearson correlation analysis. A p value < 0.05 was considered as significant. All statistics were performed with IBM SPSS Statistics (Version 28).

## Results

The analysis included 60 pwCF who utilized the Sleepiz One + device for a minimum of one month; data from two patients were excluded due to unstable signal quality (due to an unstable internet connection or because the device was not used) leaving 58 pwCF in the final analysis (52% male, mean age 32 ± 8 years, percent predicted forced expiratory volume in 1 s [ppFEV_1_] 51 ± 15) (Table 1).

**Table 1:** Participant characteristics at baseline

Parameter	Participants (*n* = 58)
Male sex, n (%)	30 (51.7)
Age, years	32 ± 8
Body mass index, kg/m²	22 ± 3
ppFEV_1_	51 ± 15
F508del homozygous, n (%)	21 (36.2)
Apnea-hypopnea index, events/h	3 ± 4
Breathing rate, cycles/min	22 ± 4
Heart rate, beats/min	66 ± 12
Epworth Sleepiness Scale score	8 ± 11

Values are mean ± standard deviation or number of participants (%)

ppFEV_1_, percent predicted forced expiratory volume in one second

The total number of home sleep recordings analyzed was 2961 (an average of 90 nights per patient). Missing nights occurred because patients forgot to switch the device on or connect hotspot, positioning was suboptimal, or because the device was not used (e.g. during hospital admission or vacation). A total of 2908 recordings met the signal duration and quality criteria.

### Respiration rate and heart rate during ETI therapy

**Fig. 1:**
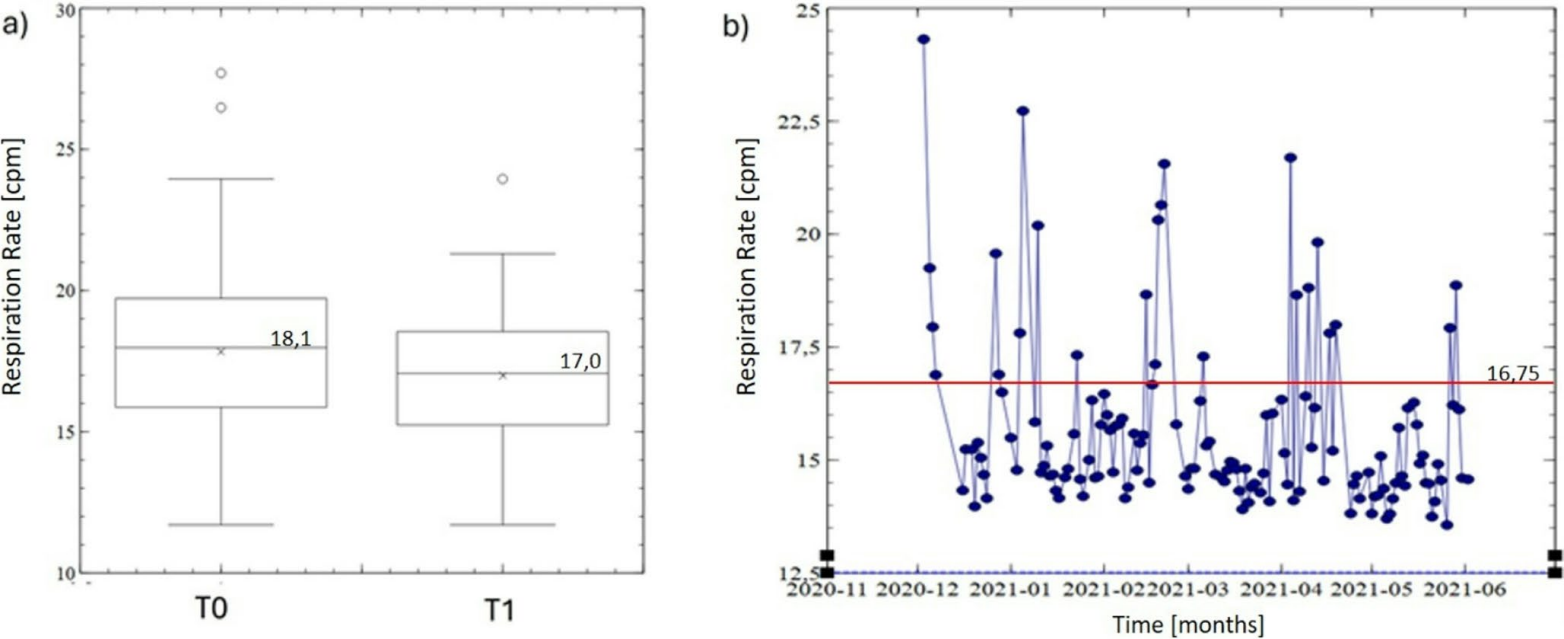
Box plot showing the mean respiration rate for all participants at baseline (T0) and 6-month follow-up (T1) (**a**) and longitudinal respiration rate data for a single patient who had an infection at the start of ETI therapy (**b**)

Estimated mean respiratory rate decreased significantly between baseline and 6 months (from 18.1 to 17.0 cycles/min; *p* < 0.0001) (Fig. 1a). A significant reduction in the respiratory rate was detected on average 1–5 days after starting ETI therapy. Some individual patients showed a short-term increase in respiratory rate after starting ETI therapy, but this was found to be due to the presence of an infection at this time (as corroborated by medical records) (Fig. 1b).

**Fig. 2:**
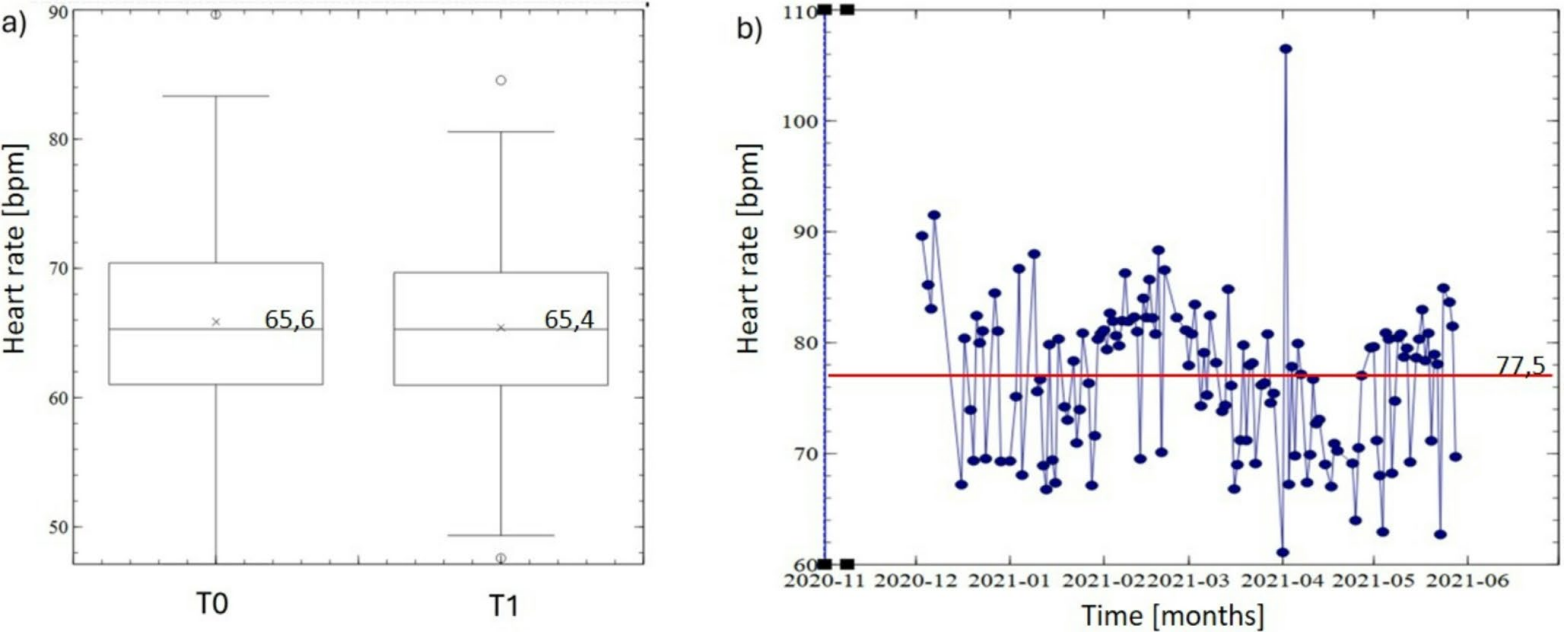
Box plot showing the mean heart rate for all participants at baseline (T0) and 6-month follow-up (T1) (**a**) and longitudinal heart rate data for a single patient who had an infection at the start of ETI therapy (**b**)

There was a slight, but non-significant, reduction in mean heart rate during ETI therapy according to Sleepiz One + data (from 65.6 to 65.4, *p* = 0.74) (Fig. 2a). This was due to differences in heart rate response to ETI therapy between patients; for example, (25%) of patients showed an unexplained increase in heart rate under ETI. Figure 2b shows an example of the longitudinal heart rate curve estimated by Sleepiz One + during ETI therapy; this patient had an infection at the time of ETI initiation and the very high values are to be regarded as outliers.

### Time in bed and sleep efficiency during ETI therapy

**Fig. 3:**
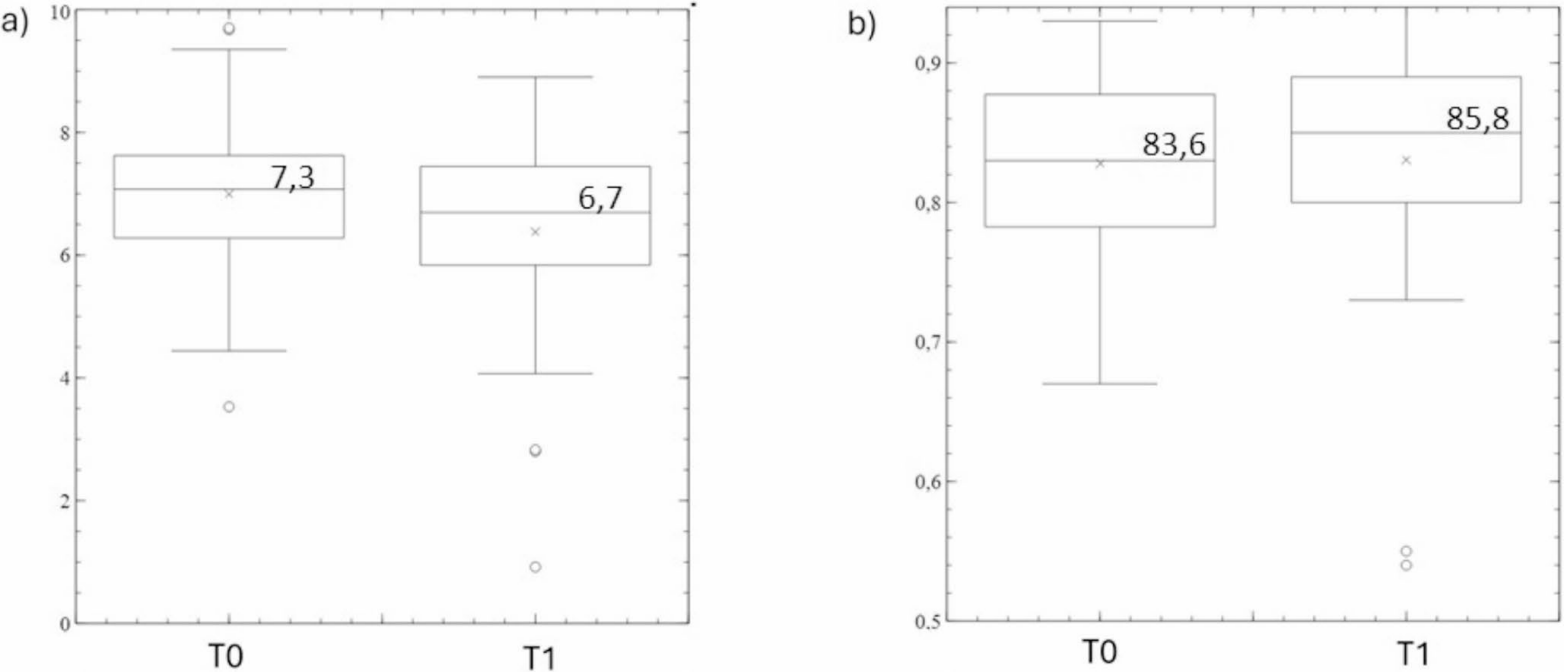
Box plots showing the time in bed in h (**a**) and sleep efficiency (**b**) for all participants at baseline (T0) and 6-month follow-up (T1)

There was a significant reduction in the time in bed estimated by Sleepiz One + from baseline to 6 months (–37 min, *p* < 0.05) (Fig. 3a). Sleep efficiency was slightly, but not significantly, higher during ETI therapy compared with baseline (+ 2.2%, *p* = 0.08) (Fig. 3b).

### Nocturnal respiratory events during ETI therapy

**Fig. 4:**
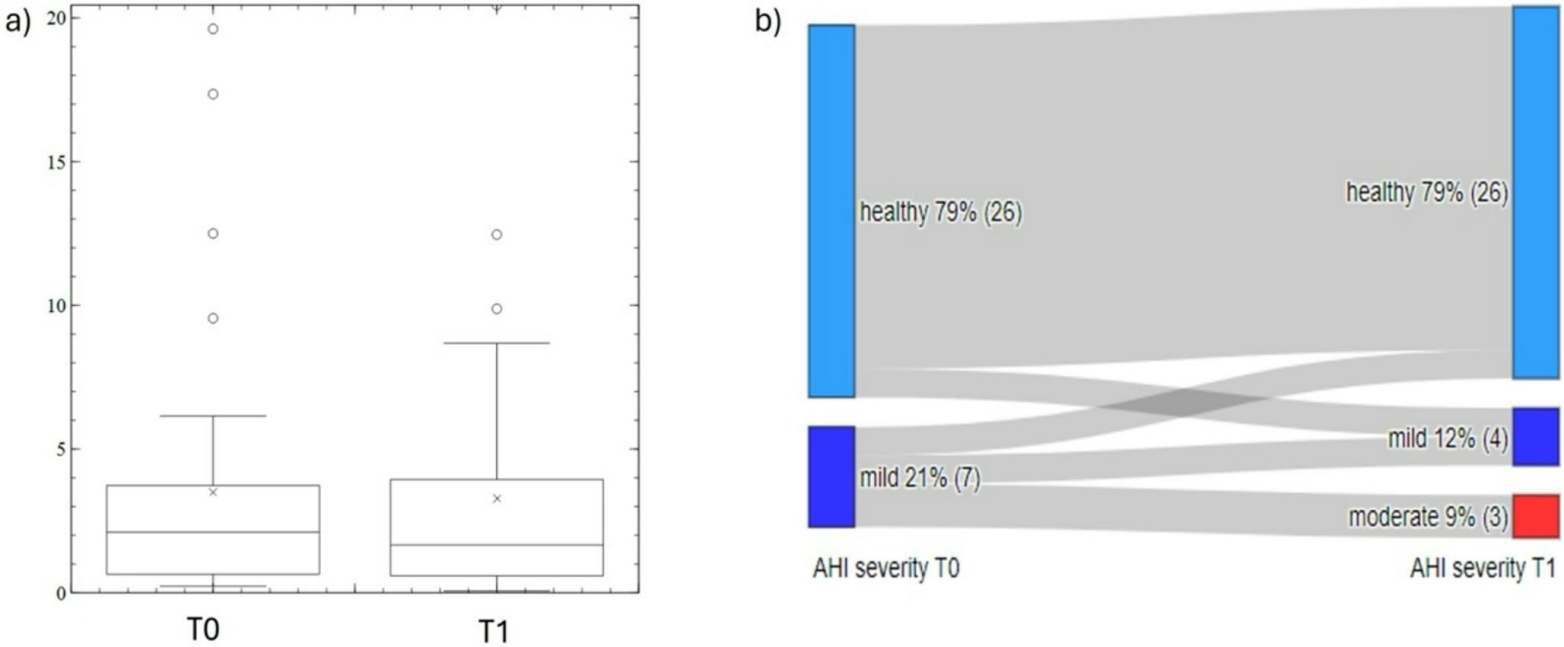
Box plot showing the apnea-hypopnea index (AHI) in events/h for all participants at baseline (T0) and 6-month follow-up (T1) (**a**) and a Sankey plot showing changes in AHI severity over time (**b**). Healthy AHI, < 5 events/h; Mild obstructive sleep apnea, AHI 5 to < 15 events/h; moderate obstructive sleep apnea, AHI 15 to < 20 event/h

There was a small but statistically nonsignificant decrease in the AHI index estimated by Sleepiz One + during ETI therapy (–3.2 events/h vs. baseline, *p* = 0.061) (Fig. 4a). A large proportion of patients had an AHI of < 5 events/h at baseline and 6-month follow-up. Seven patients had mild OSA (AHI 5 to < 15 events/h) at baseline, and this had progressed to moderate OSA (AHI 15 to < 20 event/h) at the 6-month assessment in four. For these individuals, the increase in AHI over time was significantly associated with an increase in the body mass index (BMI; *r* = 0.4, *p* < 0.05) during ETI therapy.

## Discussion

This study presents the first long-term evaluation using the Sleepiz One + contactless biomotion sensor to monitor health and sleep quality in people with cystic fibrosis (pwCF) undergoing elexacaftor/tezacaftor/ivacaftor (ETI) therapy. The device effectively captured physiological changes, including a significant reduction in respiratory rate and time in bed, supporting its potential as a tool for home-based therapy management. Reductions in respiratory rate over six months of ETI therapy align with previous findings on ETI efficacy [9, 11–13]. Temporary increases in respiratory rate correlated with infection episodes, suggesting the device’s potential utility in identifying early signs of pulmonary exacerbations, though further research is needed. Heart rate reductions were not statistically significant but consistent with prior studies.^9–10,17−16^ Sleepiz One + also detected slight improvements in sleep efficiency and a minor, non-significant decrease in apnea-hypopnea index (AHI), supporting its validated role in detecting moderate to severe OSA [27]. – [28] Although mean AHI remained stable, some participants experienced clinically relevant increases associated with weight gain—a known side effect of ETI [27–29]. This finding underscores the importance of regular sleep monitoring in pwCF with significant BMI increases during ETI therapy. The statistically significant reduction in time in bed (TiB) observed in this study aligns with previous reports of increased activity during ETI therapy [23]. Overall, Sleepiz One + demonstrated feasibility for tracking daily respiratory and sleep parameters, offering clinically relevant insights into therapy response. Its non-invasive, user-friendly design is expected to support high adherence, making it a promising tool for long-term remote monitoring in CF care.

The Sleepiz One + uses radar technology to detect breathing patterns by measuring relative changes in distance between the patient’s thorax and the device. While absolute thoracic motion may vary depending on body posture and orientation, apnea and hypopnea events are typically marked by characteristic relative changes in respiratory effort—such as reductions or cessations—that remain detectable regardless of position. Although the device does not currently classify body position explicitly, the radar signal captures dynamic respiratory features necessary for assessing obstructive sleep apnea (OSA) across all postures.

At present, the device is intended to detect and quantify respiratory events associated with sleep apnea without distinguishing between different body positions. This is in line with its current intended use, which focuses on assessing the severity of sleep-disordered breathing (e.g., via AHI estimation) independently of sleep posture. As such, further analysis of body position lies outside the scope of this study and the regulatory claims of the device.

Validation studies of the Sleepiz One + have been conducted alongside polysomnography (PSG)—the clinical gold standard for diagnosing sleep-disordered breathing—under routine conditions, where patients naturally changed sleep positions throughout the night. Despite these positional changes, the device showed strong agreement with PSG metrics, indicating that respiratory events were reliably detected regardless of posture.

While incorporating body position classification could potentially provide additional diagnostic value in the future, current validation results suggest that the lack of this feature has not negatively impacted the accuracy of the device’s OSA assessment. Therefore, body position detection is considered a possible area for future development rather than a current limitation.

The reviewer’s comment regarding the distinction between obstructive and central apneas and the potential influence of apnea subtype on AHI estimation and diagnostic accuracy is well taken and appreciated.

Currently, the Sleepiz One + is designed to detect and quantify respiratory events associated with sleep-disordered breathing, without differentiating between obstructive and central apneas. This approach aligns with the current intended use of the device, which focuses on assessing the severity of sleep-disordered breathing (e.g., via AHI estimation) rather than detailed event classification.

Since radar-based sensing captures respiratory movement patterns, it inherently detects variations that correspond to different apnea types. However, the current algorithms do not classify these patterns specifically as obstructive or central. Therefore, further analysis of classification accuracy by apnea subtype lies outside the scope of this work and the device’s regulatory claims.

Previous validation studies conducted alongside polysomnography have shown strong agreement in AHI estimation, even in the presence of a mix of apnea types as typically encountered in clinical settings. This supports the robustness of the device’s performance in real-world use, despite the absence of explicit subtype classification.

While the differentiation of apnea subtypes may be relevant in certain clinical contexts and represents a potential direction for future algorithmic development, within the current scope of use, the lack of subtype classification is not considered a limitation, but rather an area for future enhancement.

### Limitations

The main limitations of this study are the small sample size (58 pwCF) and the 6-month follow-up period. Longer observation is needed to assess the long-term feasibility and reliability of Sleepiz One + monitoring and its potential to detect infections. Data on patient acceptability and usability are also lacking, which would be important for clinical implementation. Future studies should evaluate patient satisfaction, setup simplicity, and technical challenges (e.g., device placement and connectivity), which are critical for broader implementation. Finally, results are only generalizable to adult pwCF initiating ETI therapy; further studies are needed in other populations and settings.

Another important aspect is the relatively low usage rate of the Sleepiz One + device: with 2,961 recordings from 58 patients over a period of 6 months, the average adherence rate was around 50%. This could be due to usability issues (inability to connect the device to the internet), technical problems (such as internet outages in the home environment), or fluctuations in patient adherence, which is a common problem in this patient population. The impact of such incomplete data coverage on the representativeness of longitudinal trends and the generalizability of the results must, of course, be taken into account.

Furthermore, the generalisability of our findings is limited to adults with cystic fibrosis initiating ETI therapy. Future studies are needed to validate the performance and utility of the Sleepiz One + device in broader patient populations, particularly those with diagnosed obstructive sleep apnea (OSA) or other respiratory comorbidities, where sleep-disordered breathing is common and clinically relevant.

Additionally, the Sleepiz One + device does not directly measure oxygen desaturation or sleep arousals. Similarly, body position (supine vs. nonsupine) is not recorded. However, arousals and potential sleep fragmentation may be indirectly reflected through changes in breathing patterns or interruptions in regular sleep-related respiratory rhythms. Further studies comparing Sleepiz data with full polysomnographic assessments are needed to validate such inferences.

## Conclusions

Noninvasive monitoring of physiological function and sleep using the portable, non-invasive Sleepiz One + device was found to be feasible and able to detect changes in key parameters such as the respiratory rate and AHI. Additional research is needed to determine the clinical utility of long-term monitoring and the ability to detect clinical deterioration in pwCF.

## Data Availability

The data can be shared upon reasonable and well-justified request, subject to evaluation.
